# Potential Fifth Clade of *Candida auris,* Iran, 2018

**DOI:** 10.3201/eid2509.190686

**Published:** 2019-09

**Authors:** Nancy A. Chow, Theun de Groot, Hamid Badali, Mahdi Abastabar, Tom M. Chiller, Jacques F. Meis

**Affiliations:** Centers for Disease Control and Prevention, Atlanta, Georgia, USA (N.A. Chow, T.M. Chiller);; Canisius Wilhelmina Hospital, Nijmegen, the Netherlands (T. de Groot, J.F. Meis);; Mazandaran University of Medical Sciences, Sari, Iran (H. Badali, M. Abastabar)

**Keywords:** *Candida auris*, whole-genome sequencing, typing, clades, clonal, genotyping, fungi, fungal infections, multidrug-resistant infections, antimicrobial resistance

## Abstract

Four major clades of *Candida auris* have been described, and all infections have clustered in these 4 clades. We identified an isolate representative of a potential fifth clade, separated from the other clades by >200,000 single-nucleotide polymorphisms, in a patient in Iran who had never traveled outside the country.

In the past decade, *Candida auris* has emerged in healthcare facilities as a multidrug-resistant pathogen that can cause outbreaks of invasive infections (*1*). *C. auris* has now been identified in >35 countries, many of which have documented healthcare-associated person-to-person spread (*2*). Transmission of this yeast is facilitated by its ability to colonize skin and other body sites, as well as its ability to persist for weeks on surfaces and equipment (*3*).

Whole-genome sequencing of *C. auris* has identified 4 major populations in which isolates cluster by geography (*4*). These populations are commonly referred to as the South Asian (I), East Asian (II), African (III), and South American (IV) clades. Worldwide, *C. auris* isolates continue to cluster in 1 of the 4 clades (Figure; *5**–**7*). We report an isolate representative of a fifth clade in Iran from a patient who never traveled outside that country. The patient was a 14-year-old girl in whom *C. auris* otomycosis had been diagnosed; her case was the first known *C. auris* case in Iran (*8*). 

**Figure:**
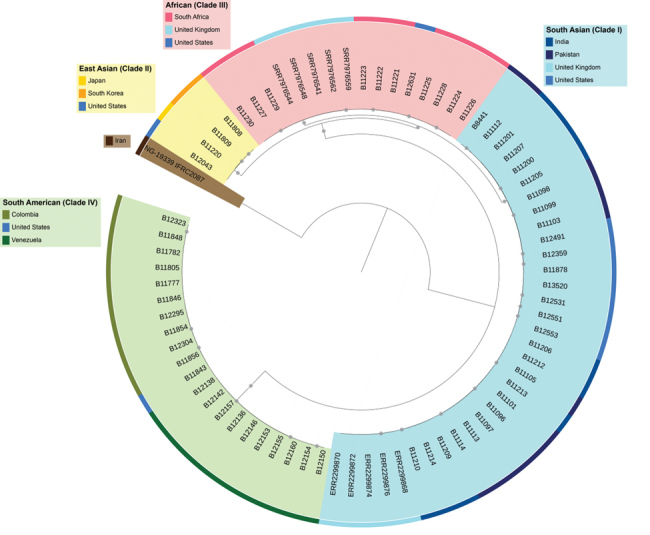
Major clades of* Candida auris*. Maximum-likelihood phylogenetic tree shows isolates from *C. auris* cases from 10 countries. Circles at nodes indicate separations with a bootstrap value >99%.

We conducted whole-genome sequencing of the isolate from Iran and 74 isolates from other countries (Appendix) and confirmed that the isolate from Iran was genetically distinct from the 4 existing clades, having a difference of >200,000 single-nucleotide polymorphisms compared with the other 4 clades. Isolates from the East Asian clade were its closest neighbors. Within the South Asian clade, isolates from *C. auris* cases in India, Pakistan, the United Kingdom, and the United States clustered together; within the East Asian clade, isolates from cases in Japan, South Korea, and the United States clustered together; within the African clade, isolates from cases in South Africa, the United Kingdom, and the United States clustered together; and within the South American clade, isolates from cases in Colombia, the United States, and Venezuela clustered together (Figure).

The C*. auris* isolate from Iran appears to represent a fifth major clade. Although this case was reported in 2018, additional cases of *C. auris* infections and colonization are thought to exist in Iran, given that challenges in diagnostic capacity in the country have probably limited the identification of more *C. auris* cases. The patient in this case was reported to have never traveled outside Iran (*8*), suggesting that this population structure might not be a result of a recent *C. auris* introduction into the country and that it might have emerged in Iran some time ago. Determining whether additional *C. auris* cases exist in Iran and whether such strains are related will help shed light on how *C. auris* emerged in Iran.

The isolate from Iran was susceptible to the 3 major classes of antifungal drugs and was cultured from ear swab specimens from the patient (*8*). *C. auris* of the East Asian clade is thought to have a propensity for the ear that is uncharacteristic of the other major clades (*9*). A recent study showed that, of 61 *C. auris* isolates obtained from 13 hospitals across South Korea during a 20-year period, 57 (93%) came from ear cultures (*10*). Although a systematic analysis has not been conducted, there are limited reports of ear infections or colonization caused by *C. auris* of the South Asian, African, or South American clades, so it is of interest that the isolate from Iran was most closely related to isolates of the East Asian clade, albeit with a difference of hundreds of thousands of single nucleotide polymorphisms. Ultimately, our discovery is a reminder that much about *C. auris* remains to be learned and underscores the need for vigilance in areas where *C. auris* has not yet emerged.

Appendix Additional information regarding a potential fifth clade of *Candida auris,* Iran, 2018.
