# Supplementary material for: Potential Fifth Clade of Candida auris, Iran, 2018
Source: Emerg Infect Dis. 2019 Sep;25(9):1780–1. doi: 10.3201/eid2509.190686 (PMC6711235; doi:10.3201/eid2509.190686)
Supplement: Appendix — Additional information regarding a potential fifth clade of Candida auris, Iran, 2018. [file 19-0686-Techapp-s1.pdf]

Article DOI: https://doi.org/10.3201/eid2509.190686

Potential Fifth Clade of *Candida auris,* Iran, 2018

Appendix

Materials and Methods

DNA Isolation

Genomic DNA was extracted from 2 day-old cultures of the Iranian *C. auris* isolate as described earlier (*1*).

Whole-Genome Sequencing

Genomic libraries were constructed and sequenced using Illumina technology (Illumina, San Diego, USA) and read length of 150 bp at Eurofins Genomics (Ebersberg, Germany). Seventy-four *C. auris* WGS sequences from NCBI were added to the analysis. FastQC and PRINSEQ was used to assess quality of read data and perform read filtering. Read data were aligned against a publically available genome sequenced on PacBio RS II using BWA. SNP variants were identified using SAMtools and filtered using the publically available SNP analysis pipeline NASP to remove positions that had less than 10x coverage, less than 90% variant allele calls, or that were identified by Nucmer as being within duplicated regions in the reference. Phylogenetic analysis and bootstrapping with 1000 iterations was performed on SNP matrices using RAxML.

Data Availability

Raw sequence read files were uploaded to the NCBI Sequence Read Archive and are publicly available under BioProject ID: PRJNA541007 (Submission ID: SUB5576192).

Reference

1. Prakash A, Sharma C, Singh A, Kumar Singh P, Kumar A, Hagen F, et al. Evidence of genotypic diversity among *Candida auris* isolates by multilocus sequence typing, matrix-assisted laser desorption ionization time-of-flight mass spectrometry and amplified fragment length polymorphism. Clin Microbiol Infect. 2016;22:277.e1–9. PubMed https://doi.org/10.1016/j.cmi.2015.10.022
